# The Relationship Between Dietary Fiber Intake and Blood Pressure Worldwide: A Systematic Review

**DOI:** 10.7759/cureus.46116

**Published:** 2023-09-28

**Authors:** Vitrag N Tejani, Sukhmeet S Dhillon, Nanush Damarlapally, Nia Uswanti Binti Usman, Tanusha Winson, Prithvi Basu Roy, Binay K Panjiyar

**Affiliations:** 1 Internal Medicine, Parul Institute of Medical Sciences and Research, Parul Sevashram Hospital, Parul University, Vadodara, IND; 2 Pharmacology, Dr. N. D. Desai Faculty of Medical Science and Research, Nadiad, IND; 3 Internal Medicine, Baba Farid University of Health Sciences, Patiala, IND; 4 Health Sciences, Coleman College of Health Sciences, Houston, USA; 5 Internal Medicine, Universitas Brawijaya, Kajang, MYS; 6 Medicine, Asian Institute of Medicine, Science and Technology (AIMST) University, Bedong, MYS; 7 Cardiology, KPC (Kali Pradip Chaudhuri) Medical College and Hospital, Kolkata, IND; 8 Cardiology, Harvard Medical School, Boston, USA; 9 Internal Medicine, California Institute of Behavioral Neurosciences & Psychology, Fairfield, USA

**Keywords:** cardiovascular health, diastolic blood pressure, systolic blood pressure, global health, nutrition, hypertension, dietary fiber, blood pressure

## Abstract

Cardiovascular diseases (CVDs) are a significant global health concern, necessitating effective preventive measures. Dietary fiber has gained attention as a potential cardiovascular risk factor modifier. Although its effects on various CVD risk markers such as cholesterol levels and blood glucose levels have been explored, the relationship between dietary fiber and blood pressure remains somewhat elusive across the different studies conducted worldwide. In this systematic review, we conducted an extensive analysis of recent research from a global perspective, aiming to elucidate the relationship between dietary fiber intake and blood pressure. From an initial pool of more than 24,500 articles retrieved from PubMed and Google Scholar, we rigorously selected 11 studies published in the last decade (post-2013) to ensure up-to-date insights. These selected studies encompass diverse populations from different regions worldwide, allowing for a comprehensive global assessment. Our analysis revealed a positive overall impact of increased dietary fiber intake on blood pressure levels. Despite variations in study parameters, consistent trends were observed across multiple continents. This systematic review underscores the potential of dietary fiber intake to reduce blood pressure and improve cardiovascular health globally. This review serves as a global analysis and updates on the developments about the potential association between dietary fiber and blood pressure levels. While the findings are promising, further research is essential to elucidate underlying mechanisms and ensure global consistency. Collaborative efforts and ongoing investigation are crucial for harnessing the cardiovascular benefits of dietary fiber and addressing the worldwide burden of hypertension.

## Introduction and background

With each passing day, the burden of cardiovascular disease (CVD) on global health escalates, underscoring the urgency of effective preventive strategies. Amid this relentless battle, preventive measures that seamlessly integrate into daily routines take center stage. In this systematic review, we embark on a journey through the intricate landscape of cardiovascular risk factors and dietary intervention, with a specific focus on dietary fiber. While dietary fiber has shown promising effects by modifying various CVD risk markers, the enigmatic connection between dietary fiber and blood pressure on a global scale remains a captivating puzzle that demands meticulous investigation.

CVDs continue to take a staggering toll on global mortality and morbidity, resulting in countless premature deaths each year. To emphasize the gravity, one person succumbs to a CVD-related event every 40 seconds in the USA [1]. Translating these findings to a global scale, approximately 20.5 million people died of CVDs worldwide in 2021, accounting for almost one-third of all deaths that year [2]. Against this grim backdrop, our global imperative must revolve around the urgent quest for efficient preventive approaches alongside curative interventions.

Age, gender, and family history are non-modifiable risk factors for CVDs that lie beyond our control. However, there exist modifiable risk factors such as a sedentary lifestyle, an unhealthy diet, physical inactivity, and so on that warrant our unwavering attention [3,4]. Among these modifiable variables, the promotion of a heart-healthy diet emerges as a potent and cost-effective counterstrategy, making it a compelling primary target for CVD prevention [5].

Dietary fibers are non-digestible carbohydrates that offer various health benefits [6]. It has been proven in various clinical studies that dietary fiber lowers cholesterol levels in humans [7]. Additionally, there have been studies that have shown an inverse relationship between higher dietary fiber consumption and glucose levels in diabetics (yet another risk factor for CVD) [8]. These collective findings imply that dietary fiber could be a promising ally for the prevention and management of CVDs at global levels.

Blood pressure, a vital physiological parameter, serves as a critical indicator of cardiovascular health [9]. Systolic blood pressure (SBP) and diastolic blood pressure (DBP) reflect the force exerted by circulating blood against arterial walls during the heart's contraction and relaxation phases, respectively. Optimal blood pressure levels are crucial for efficient organ perfusion, oxygen supply, and delivery of nutrients to tissues while efficiently eliminating waste products [10]. The delicate balance in blood pressure regulation is fundamental to overall cardiovascular well-being, representing the intricate interplay of multiple physiological processes within the human body.

Several proposed mechanisms shed light on how dietary fiber might impact blood pressure. One such mechanism is attributed to soluble fibers, including beta-glucans and pectins, which can form viscous gels in the gastrointestinal tract, potentially slowing down carbohydrate digestion and glucose absorption [11]. Such an effect could potentially modulate insulin sensitivity and improve glycemic control, thereby influencing blood pressure. Secondly, the fermentation of dietary fiber by gut microbiota results in the synthesis of short-chain fatty acids (SCFAs), particularly propionate and butyrate, which have been linked to blood pressure reduction. SCFAs might exert vasodilatory effects through the relaxation of blood vessel walls or modulation of the renin-angiotensin-aldosterone system (RAAS) [12]. Additionally, the ability of dietary fiber to modulate the composition of the gut microbiota may be a factor in blood pressure regulation, as there is a growing body of evidence linking gut dysbiosis to hypertension [13].

While systematic reviews and meta-analyses conducted in the USA have yielded promising results regarding the effects of dietary fiber on blood pressure [14,15], there is a pressing need for a comprehensive global investigation. This study aims to answer the question: 'What is the impact of dietary fiber intake on blood pressure levels in diverse countries?' By examining the most recent studies from various regions, our overarching objective is to inform strategies for combating CVDs on a global scale. In this revision, the research question is clearly stated: "What is the impact of dietary fiber intake on blood pressure levels in diverse countries?"

While dietary fiber has demonstrated promise in influencing blood pressure, past research has revealed variations in study designs, population demographics, and the specific types of dietary fiber investigated. Some studies have shown exclusive effects on DBP, while others have focused on SBP, highlighting the complexity of this relationship. Given the critical role of blood pressure in CVD and its wide-reaching implications for cardiovascular health, there is a need to consolidate global knowledge from studies worldwide. With this objective in mind, our systematic review comprehensively explores the potential link between dietary fiber intake and blood pressure levels on a global scale, aiming to identify trends in this significant connection.

## Review

Methods 

**Figure 1:**
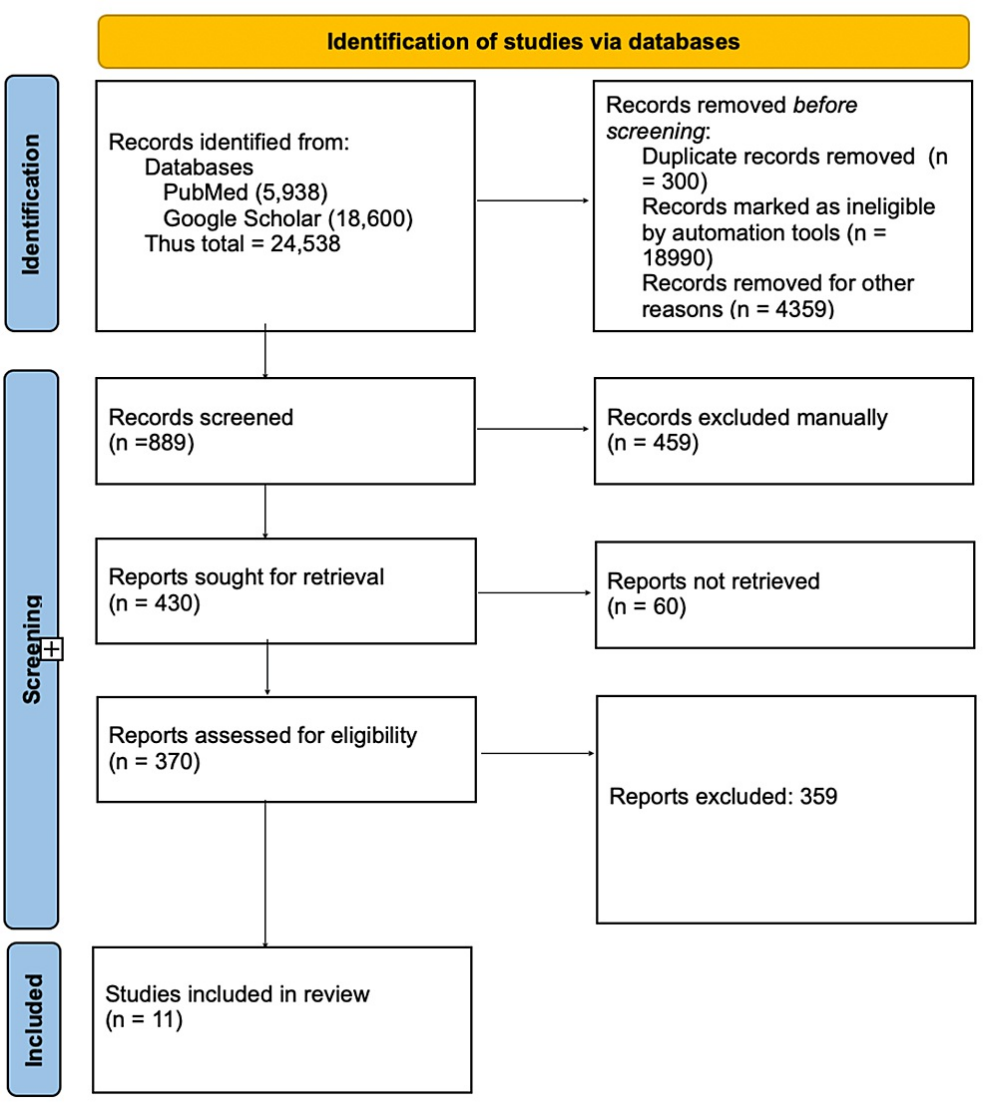
PRISMA flow diagram illustrating the search strategy and study selection process for the systematic review PRISMA, Preferred Reporting Items for Systematic Reviews and Meta-Analyses

The clinical studies that link dietary fiber consumption to its impact on blood pressure are the primary focus of this review. Animal studies and articles that only discussed the methodology without providing clinical data were excluded. The review complies with the Preferred Reporting Items for Systematic Reviews and Meta-Analyses (PRISMA) guidelines for 2020 (Figure 1) and relies solely on information gathered from articles that have already been published, obviating the need for ethical review [16].

Systematic Literature Search and Study Selection

We used PubMed and Google Scholar to thoroughly search for pertinent publications. On PubMed, we looked for studies that were cited in reviews, editorials, and commentaries. Even so, we kept looking for more studies that matched our inclusion criteria.

We independently evaluated a list of abstracts for inclusion according to predetermined standards. The criteria included higher fiber intake interventions and those whose effects on physiologic parameters had been studied and the same observed on blood pressure. Review articles and animal studies were not included. A dual review was performed by two reviewers, and any disagreements were settled through conversation.

Inclusion and Exclusion Criteria

To meet the objectives of our study, we established precise standards for the inclusion and exclusion of participants. Table 1 provides a summary of our criteria.

**Table 1:** Criteria adopted during the literature search process *The Population (P), Intervention (I), Control (C), and Outcome (PICO) criteria utilized were as follows: P: Adult I: Any type of increased fiber intake been achieved through some dietary modification C: As compared to usual dietary habits or a lower dietary fiber intake O: Any change in blood pressure levels or not

Inclusion criteria	Exclusion criteria
Human studies only	No animal studies were included
From 2013 to 2023	Non-English
English text	Age < 19 years
Gender - all	Papers that needed to be purchased
Age > 19 years of age	
Free full papers	
PICO criteria*	

Search Strategy

The search was conducted on PubMed and Google Scholar Libraries, using relevant keywords. We had two major concepts to look upon across the studies with a variety of demographics: dietary fiber and blood pressure.

Keywords identified for dietary fiber were: “dietary fiber,” “fiber intake,” “dietary fiber sources,” “fiber rich food,” “fiber consumption.” The medical subject heading (MeSH) term we identified was “dietary fiber”[Mesh]. Similarly, we also used an array of keywords for the blood pressure so as to receive maximal possible relevant results.

A thorough search strategy was created using the MeSH approach for PubMed and next, Google Scholar, as shown in Table 2.

**Table 2:** Search strategy, search engines used, and the number of results displayed.

Database	Search strategies	No. of articles
PubMed filters applied: free full text, case reports, clinical trial, meta-analysis, randomized controlled trial, review, systematic review, published in the last 10 years, included humans, in English, adults aged 19+ years	"Dietary Fiber"[Mesh] OR Dietary Fiber OR Fiber Intake OR Dietary Fiber sources OR Fiber rich food OR Fiber Consumption OR "Blood Pressure"[Mesh] OR “systolic blood pressure” OR “Diastolic blood pressure”	5,938
	"Dietary Fiber"[Mesh] OR Dietary Fiber OR Fiber Intake OR Dietary Fiber sources OR Fiber rich food OR Fiber Consumption	860
	"Blood Pressure"[Mesh] OR “systolic blood pressure” OR “Diastolic blood pressure”	5,078
	("Dietary Fiber"[Mesh] OR Dietary Fiber OR Fiber Intake OR Dietary Fiber sources OR Fiber rich food OR Fiber Consumption AND ((y_10[Filter]) AND (ffrft[Filter]) AND (casereports[Filter] OR clinicaltrial[Filter] OR meta-analysis[Filter] OR randomizedcontrolledtrial[Filter] OR review[Filter] OR systematicreview[Filter]) AND (humans[Filter]) AND (english[Filter]) AND (alladult[Filter]))) AND ("Blood Pressure"[Mesh] OR "systolic blood pressure" OR "Diastolic blood pressure" AND ((y_10[Filter]) AND (ffrft[Filter]) AND (casereports[Filter] OR clinicaltrial[Filter] OR meta-analysis[Filter] OR randomizedcontrolledtrial[Filter] OR review[Filter] OR systematicreview[Filter]) AND (humans[Filter]) AND (english[Filter]) AND (alladult[Filter])))	48
Google Scholar	"dietary fiber" AND "blood pressure"	18,600

Quality Appraisal

We used a variety of quality assessment tools to ensure the dependability of the papers we selected. For randomized clinical trials used in systematic reviews and meta-analyses, we used the PRISMA checklist and Cochrane bias tool assessment. Using the Newcastle-Ottawa Tool Scale, non-randomized clinical trials were assessed. We assessed the quality of qualitative studies, as shown in Table 3, using the critical appraisal skills program (CASP) checklist.

**Table 3:** Quality appraisal tools utilized PRISMA, Preferred reporting items for systematic reviews and meta-analyses; RCT, randomized controlled trial

Quality appraisal tool	Studies
Cochrane bias tool assessment	RCTs
Newcastle-Ottawa tool	Non-RCT and observational studies
PRISMA checklist	Systematic reviews

Results

After skimming PubMed and Google Scholar, our two selected databases, we had 24,538 articles. We then applied specific criteria, including the PICO (Population, Intervention, Control, and Outcome). In Google Scholar, we reviewed the first 10 pages of Google Scholar results. Then, collectively, from both resources, we were able to narrow it down to 370 records. Articles lost included duplicates, those lost to automation, and a few lost during retrieval. We then manually excluded those articles after careful review that did not align with our purpose. Throughout the search, we were looking for a variety of demographics so as to derive a global trend. We were able to have 11 papers for our thorough systematic review after a rigorous quality check on them. Key findings from each of them are compiled in Table 4.

**Table 4:** Summary of the results of the selected papers CI, confidence interval; DBP, diastolic blood pressure; GA, gum Arabic; LDL, low-density lipoprotein; MD, mean difference; RCT, randomized controlled trial; SBP, systolic blood pressure; UAE, United Arab Emirates; WGPF, wine grape pomace flour

Author/year	Country	Study design	Database used	Conclusion
Reynolds et al./2022 [17]	Multiple countries	Systematic Review	PubMed	In hypertensive individuals, it lowers SBP by 4.3 mm Hg and DBP by 3.1 mm Hg. It also lowers LDL.
Hartley et al./2016 [18]	Multiple countries	Systematic review	PubMed	There was a statistically significant lowering in DBP, whereas SBP lowering was not statistically significant (DBP mean difference = -1.77 mm Hg; 95% CI: -2.61 to -0.92).
Huang et al./2017 [19]	Multiple countries	Meta-analysis	PubMed	The study found that chitosan administration did not significantly SBP. However, subgroup analyses indicated that chitosan consumption significantly reduced DBP in shorter-term interventions (p=0.006) and at higher dosages.
Urquiaga et al./2015 [20]	Chile	RCT	PubMed	The study showed a significant decrease in both SBP and DBP after consuming WGPF for 16 weeks. However, it is important to note that the study had a relatively small sample size and focused only on males, which may limit the generalizability of the findings.
Babiker et al./2018 [21]	Sudan	RCT	PubMed	SBP significantly decreased by 7.6% in the GA group and by 2.7% in the placebo group from baseline. However, there were no significant changes observed in DBP. GA consumption at a dose of 30 g/day for three months may have a beneficial effect on SBP in individuals with type 2 diabetes.
Jarrar et al./2021 [22]	UAE	RCT	PubMed	GA supplementation in adults at risk of metabolic syndrome has shown positive effects on blood pressure, indicating its potential as a beneficial adjunct in managing cardiovascular risk factors.
Tessari and Lante et al./2017 [23]	Italy	RCT	PubMed	Bread containing high fiber was used as an intervention in type 2 diabetics. There was a significant lowering of glycated hemoglobin but statistically significant differences were not observed in SBP or DBP.
Fechner et al./2014 [24]	Germany	RCT	PubMed	The lupin kernel fiber period resulted in a significant decrease in SBP compared to the baseline and the low-fiber control diet period. Both the lupin fiber and citrus fiber periods led to lower SBP compared to the control diet period. There were no substantial changes in DBP observed during the study.
Aljuraiban t al./2015 [25]	USA	Cross-sectional studies	Google Scholar	A higher intake of insoluble dietary fiber was associated with a statistically significant decrease in DBP and SBP. The same effect was not observed with soluble dietary fiber.
Khan et al./2018 [26]	Multiple countries	Meta-analysis	Google Scholar	Viscous soluble fiber reduced SBP (MD = −1.59 mm Hg [95% CI: −2.72, −0.46]) and DBP (MD = −0.39 mm Hg [95% CI: −0.76, −0.01]) compared to controls.
Xue et al./2021 [27]	China	RCT	Google Scholar	In a three-month study, dietary fiber supplementation was investigated in hypertensive patients, revealing significant reductions in both office and ambulatory blood pressure compared to a control group. The intervention also led to changes in gut microbiota composition, including increased Bifidobacterium and Spirillum levels.

Discussion

The rising prevalence of CVDs worldwide is a pressing public health concern, given that CVDs currently stand as the leading cause of global mortality. As we strive to address the risk factors contributing to CVDs, it is crucial to focus on modifiable factors. Among these, hypertension stands out as a significant risk factor. In this systematic review, our aim is to investigate the worldwide relationship between dietary fiber intake and blood pressure levels. Our objective is to discern global patterns in dietary fiber consumption and its impact on blood pressure. By doing so, we seek to illuminate potential strategies for preventing and managing hypertension on a global scale. In the course of this discussion, we seek to answer a fundamental question: “Does a higher intake of dietary fiber have a measurable influence on blood pressure levels worldwide?”

As we explored the vast body of research, our combined discoveries started to reveal an encouraging story - eating more dietary fiber might be a positive factor in managing blood pressure. However, the journey to this insight is filled with complexities due to differences in how studies were conducted, the kind of interventions used, and the diverse groups of people studied. Therefore, more research is needed to confirm these findings and understand exactly how dietary fiber affects blood pressure. With this broad view in mind, let us dive into individual studies that have shed light on this intricate connection.

Underlying Mechanisms

There are several underlying mechanisms that explain the interplay of having a lower BP when dietary fiber intake is higher. Reynolds et al. have explained several underlying mechanisms in their study [17]. They have explained that the hypolipidemic effect of fibers improves the elasticity of blood vessels. This works to bring down systemic vascular resistance. They noted that nitric oxide is increased with higher fiber intake [17]. Nitric oxide being a vasodilator contributes to blood pressure management. There is an improvement in insulin sensitivity with fibers that they have mentioned. This too plays a minor role in reduction of blood pressure [17]. They also postulated that higher fiber intake may help with weight reduction. This may again help to lower blood pressure [17].

Strengths and Limitations of Studies From Across the World

The effectiveness of increased consumption of dietary fiber for the primary prevention of CVDs was examined in a systematic review by Hartley et al., and they found that it was helpful in lowering DBP and low-density lipoprotein levels [18]. The subjects in the randomized controlled trials (RCTs) they included were from the USA, Japan, China, Australia, and European countries and thus have good generalized applicability. However, because their review did not examine data on mortality and the intervention duration varied from three to six months, effects over longer term is still an unexplored area [18]. Only then can we make a statement about its impact on clinical practice for primary prevention. In our systematic review, we will also explore and try to determine whether any particular fiber type has a higher impact. We will also look to see if the dosage and duration of the study had different impacts.

In an attempt to look at the above factors, we landed first on the meta-analysis of eight RCTs by Huang et al., which studied the effect of chitosan (an insoluble fiber) on blood pressure [19]. They went on to conduct a subgroup analysis to explore the effects of duration and dosage on blood pressure. Overall, chitosan administration did not significantly lower SBP or DBP [19]. However, subgroup analyses indicated that chitosan consumption significantly reduced DBP in shorter-term interventions and at higher doses. They found that higher-dose chitosan (more than 2.4 g/day) and shorter-term interventions (less than 12 weeks) resulted in a significant reduction in DBP (weighted mean difference: -3.50 mm Hg; 95% CI: -6.40 to -0.59; p < 0.02) [19]. The number of available RCTs with higher doses and shorter interventions was limited; therefore, generalized applicability is still limited, even though the study had RCTs from a wide range of countries [19].

Wine grape pomace flour (WPGF), a potent fiber-antioxidant-containing ingredient and another rich source of insoluble fiber, was used as an intervention by Urquiaga et al. in their RCT in Chile [20]. Consumption of WGPF significantly lowered blood pressure levels along with boosted antioxidant defenses, lowered oxidative protein damage, and postprandial insulin levels, as well as glycemia and other indicators of oxidative stress [20]. This holistic impact is also partially due to the antioxidant properties of WPGF. The small sample size, short duration of intervention, and the exclusive focus on males in this study limit generalized applicability [20]. Also, the study did not provide specific numerical values for blood pressure reduction, making the assessment of the magnitude of the observed effect difficult.

There are many studies that looked at this relationship and had an intervention comprising soluble fibers. The next few studies focus on developments related to soluble fiber consumption and its impact on blood pressure. In Sudan, Babiker et al. looked into the impact of supplementing gum Arabic (GA), a soluble fiber, and its effect on type 2 diabetes patients' blood pressure and anthropometric obesity markers [21]. They noticed a significant reduction in SBP (7.6%), whereas DBP was not noted to be significantly reduced [21]. Jarrar et al. used GA supplementation as an intervention in the United Arab Emirates to observe its effect on metabolic syndrome parameters, and, thus, made an observation of its effect on blood pressure [22]. According to them, GA improved blood pressure, and thus we get one more compelling candidate for long-term studies in the form of GA [22].

The systematic review and meta-analysis by Khan et al. investigated the effects of supplemental viscous soluble fiber on blood pressure [26]. The research showed that both SBP and DBP were reduced slightly but significantly [26]. The substantial heterogeneity in their results suggests that further research is needed to better understand the effects of different types of viscous fibers and identify potential sources of variation in the outcomes [26]. This leads to an interest in having studies that further delve into the types of fibers and their effects on blood pressure.

Another piece of evidence comes from China, where Xue et al. investigated the effects of oat bran (soluble fiber) on blood pressure and gut microbiota [27]. Their research demonstrated significant blood pressure drops. The study was only conducted over a three-month period and had a rather small sample size (50 participants), which could limit how broadly the findings can be applied [27].

In Germany, Fechner et al.'s research showed the possible cardiovascular advantages of lupin kernel fiber [24]. Their research highlighted the function of SCFAs in mediating these effects of lowering blood pressure and showed how dietary fiber might modify cardiovascular risk factors, including its hypolipidemic effects [24]. In Italy, Tessari and Lante looked into how a beta glucan-rich (soluble fiber) functional bread affected type 2 diabetics' ability to control their metabolism. They found that blood pressure levels had not changed significantly [23]. This highlights the importance of thorough studies that examine the complex interactions between dietary fiber and numerous health outcomes.

Aljuraiban et al. investigated this promising direction in the face of such a strong demand for research into the impact of various fiber types on blood pressure [25]. The paper specifically focused on total, insoluble, and soluble fiber, and offered information regarding the relationship between dietary fiber intake and blood pressure. Because of the use of data from the INTERMAP (INTERnational study on MAcro/micronutrients and blood Pressure) study, a sizable international study, the results are more generally applicable [25]. The validity of the findings was strengthened by the analysis' adjustment for various dietary and lifestyle confounders. However, the cross-sectional design of the study makes it difficult to establish cause-and-effect relationships. Also, it becomes difficult to assess the long-term effects of fiber intake on blood pressure from a cross-sectional study. The study emphasizes the value of taking into account various fiber types. They noted that insoluble fiber shows a stronger association in lowering blood pressure as compared to soluble fiber in the study [25].

The meta-analysis and systematic review by Reynolds et al. concluded that SBP has experienced significant reductions. DBP was not reduced according to them [17]. The study included a large number of participants from multicountry cohorts and trials, providing robust evidence for the benefits of higher fiber diets on blood pressure [17]. However, what we noticed and what they acknowledged was the high heterogeneity in the trials included. Higher heterogeneity may indicate that factors beyond high fiber intake might have an impact too [17].

The systematic review of studies examining the relationship between dietary fiber intake and blood pressure levels revealed a consistent trend across diverse regions and populations. Increased consumption of dietary fiber, whether soluble or insoluble, generally exhibited a positive impact on lowering blood pressure. Notable reductions in DBP were observed in studies involving insoluble fibers such as chitosan and WPGF, particularly in shorter-term interventions and at higher doses [19,20]. On the other hand, soluble fibers such as GA and viscous soluble fiber showed promise in reducing SBP and DBP, though substantial heterogeneity in outcomes necessitates further investigation into different fiber types [21,22,26]. Oat bran, another soluble fiber source, demonstrated significant blood pressure reduction, but limited sample size and study duration may restrict broader applicability [27]. Research on lupin kernel fiber highlighted SCFAs' role in mediating blood pressure-lowering effects [24]. However, a beta glucan-rich functional bread did not significantly affect blood pressure, emphasizing the intricate nature of dietary fiber interactions with health outcomes [23].

In conclusion, the body of evidence from a diverse range of studies, spanning geographical regions and encompassing various fiber types, consistently points to the positive impact of increased dietary fiber intake on reducing blood pressure levels. While the specific mechanisms at play may vary, and the magnitude of effects may differ based on factors such as study duration, fiber type, and dosage, the overall trend is clear. This trend holds true for individuals with conditions such as type 2 diabetes, metabolic syndrome, and hypertension, as well as for broader population groups. However, it is essential to acknowledge the need for further research to establish optimal doses and long-term effects, translating these findings into practical recommendations for public health and clinical practice. These studies collectively highlight the potential of dietary fiber as a cost-effective strategy in the global effort to manage blood pressure and mitigate the burden of CVDs.

Implications for Public Health

The implications of our findings reach beyond individual health to global public health. Our review suggests that promoting increased dietary fiber intake could be a cost-effective strategy for managing blood pressure and reducing the burden of hypertension. Encouraging fiber-rich diets, especially among at-risk populations, could significantly reduce hypertension-related health issues. Policymakers should consider dietary fiber promotion as part of comprehensive strategies to combat hypertension and its associated societal and economic costs.

Implications for Clinical Practice

The findings from this systematic review on dietary fiber intake and its impact on blood pressure have significant implications for clinical practice. Healthcare professionals can incorporate this knowledge into patient counseling and recommendations, especially for individuals at risk of or diagnosed with hypertension. Encouraging patients to increase their dietary fiber intake, whether through whole grains, fruits, vegetables, or fiber-rich supplements, can be a practical and relatively simple intervention to help manage blood pressure. Clinicians may also consider tailoring fiber recommendations based on individual patient profiles, including their existing medical conditions and dietary preferences. Additionally, healthcare providers can play a vital role in educating patients about the different types of fiber and their potential benefits in blood pressure management, helping individuals make informed dietary choices. Overall, these findings underscore the importance of dietary fiber as a modifiable factor in hypertension prevention and offer a valuable tool for clinicians in their efforts to improve patient cardiovascular health.

Gaps in Knowledge and Future Research Questions

Future research in the field of dietary fiber and blood pressure should prioritize addressing critical knowledge gaps and refining study designs. An essential focus for future investigations is an exploration of the long-term effects of sustained dietary fiber intake on blood pressure. While existing studies offer valuable insights, understanding the enduring impact of dietary fiber over extended periods is crucial for comprehensive recommendations. Additionally, researchers should prioritize conducting studies that encompass diverse populations, as many previous investigations have been region-specific, potentially limiting the generalizability of findings. To establish more precise dietary recommendations, it is imperative to investigate the dose-response relationship between dietary fiber intake and blood pressure reduction. Moreover, while several potential mechanisms have been proposed, further studies are needed to elucidate the precise biological pathways through which dietary fiber influences blood pressure. Research efforts should also focus on differentiating the effects of various fiber types, including soluble and insoluble fiber, to provide more targeted dietary guidance. As research advances, the translation of these findings into practical dietary guidelines for healthcare professionals in clinical settings becomes crucial. Lastly, policymakers should consider implementing public health strategies that promote increased dietary fiber intake, recognizing it as a cost-effective approach to managing hypertension and reducing the associated health and economic burdens.

Limitations

We acknowledge several limitations in our literature review. Firstly, we focused on English publications from the last 10 years, which may introduce a potential bias toward more recent studies. Secondly, we restricted our analysis to studies that included participants aged at least 19 years old, which may limit the generalizability of our findings to younger populations. Additionally, we did not include narrative reviews in our analysis, focusing solely on original research articles. One notable limitation is that we exclusively considered articles with free full access, potentially excluding valuable studies behind paywalls or requiring purchase. This approach could introduce a selection bias, as freely accessible studies may differ in their findings or methodologies from those with restricted access. Furthermore, our review was limited to English-language papers, which may have led to the omission of relevant research published in other languages. In our systematic review, we encountered studies with shorter durations, and the presence of heterogeneity among the included studies poses a challenge in drawing definitive conclusions. The diverse types of dietary fiber involved, variations in dosage, and potential genetic associations across different populations represent important factors that warrant further exploration in future research. In conclusion, while our systematic review provides valuable insights into the relationship between dietary fiber intake and blood pressure levels, these limitations underscore the need for continued investigation and consideration of a broader range of studies to draw more comprehensive and robust conclusions.

## Conclusions

In conclusion, our systematic exploration of global dietary fiber intake trends and their impact on blood pressure presents a promising narrative. Across continents and among diverse populations, an increased dietary fiber intake shows a consistent and positive association with reduced blood pressure levels. This trend remains significant for individuals with various health conditions, including type 2 diabetes, metabolic syndrome, and hypertension. Nevertheless, the complexity of this relationship becomes apparent when we consider the diversity in research designs, intervention dosages, and types of dietary fiber used across studies. Consequently, while the potential benefits of dietary fiber on blood pressure are evident, further research is needed. Future studies should endeavor to pinpoint optimal dosages, intervention durations, and specific fiber varieties to maximize their impact. Furthermore, given the global burden of hypertension and its substantial contribution to CVDs, these findings have profound implications for public health policies and clinical practices. Promoting increased dietary fiber intake as a straightforward and cost-effective preventive measure can significantly contribute to global efforts aimed at reducing the prevalence of hypertension and mitigating related cardiovascular risks.
